# Illness perceptions in people with obsessive-compulsive disorder; A qualitative study

**DOI:** 10.1371/journal.pone.0213495

**Published:** 2019-03-20

**Authors:** Rebecca Pedley, Penny Bee, Alison Wearden, Katherine Berry

**Affiliations:** 1 Division of Psychology and Mental Health, School of Health Sciences, Faculty of Biology, Medicine and Health, Manchester Academic Health Science Centre, The University of Manchester, Manchester, United Kingdom; 2 Division of Nursing, Midwifery and Social Work, School of Health Sciences, Faculty of Biology, Medicine and Health, Manchester Academic Health Science Centre, The University of Manchester, Manchester, United Kingdom; 3 Division of Psychology and Mental Health, School of Health Sciences & Manchester Centre for Health Psychology, Faculty of Biology, Medicine and Health, Manchester Academic Health Science Centre, The University of Manchester, Manchester, United Kingdom; Victoria University of Wellington, NEW ZEALAND

## Abstract

**Background:**

Obsessive-compulsive disorder (OCD) is a serious mental health problem that causes significant impairment and reduced quality of life. Though some substantially benefit from psychological therapies, a substantial proportion of people with OCD disengage from treatment or fail to benefit. Theoretical models such as the Common-Sense Model posit that our management of physical illness depends on our perceptions about the condition. Identifying how people with OCD perceive their condition could lead to important insight that would improve treatment of OCD.

**Objectives:**

To identify and characterise the illness perceptions of people with OCD.

**Method:**

Transcribed semi-structured interviews exploring the illness perceptions of 16 people with OCD were analysed using thematic analysis.

**Results:**

In some cases, identification of symptoms was hindered by a failure to interpret experiences as ‘symptoms’. Instead, these individuals interpreted symptoms as a personality quirk, or as evidence that they had become deviant. Perceptions of the condition as ‘part’ of the self contributed to views of OCD as permanent. Individuals were concerned about the impact of OCD on friends and family and attempted to minimise its consequences, for example by concealing symptoms from their children, who they feared could acquire OCD.

**Conclusion:**

Applying a theoretical model of physical health understanding to OCD yielded novel insights, with important implications for support and treatment. To enable early help-seeking and rapid diagnosis, public and professional knowledge about OCD should be extended beyond ‘washing and checking’ to the less widely known OCD-subtypes, such as fear of causing harm. It may be important to identify and challenge views of OCD as permanent early in the course of treatment to maximise engagement. Management of OCD should also address the burden of living with OCD in a family context. Further research to test whether these perceptions lead to coping responses and outcomes in OCD is now needed.

## Introduction

Obsessive-compulsive disorder (OCD) is a mental health condition that affects approximately 2% of the population in their lifetime [1, 2]. People living with OCD experience obsessions (recurring unwanted thoughts, images or impulses) and/or compulsions (repetitive physical behaviours, such as checking or mental acts) [3]. Individuals with OCD perform these acts to provide themselves with reassurance, to prevent a feared event and/or reduce their distress or anxiety [3]. People living with OCD can experience severe ‘role’ impairment [2] and reduced quality of life [4]. In the domain of social relationships, quality of life scores are lower than those observed in other mental or physical health problems [4].

In recent decades, significant progress has been made in developing effective psychological and pharmacological treatments for OCD [5, 6]. In the UK, the National Institute for Health and Care Excellence (NICE) has recommended cognitive behaviour therapy (CBT) with exposure and response prevention (ERP) as the first-line treatment for OCD [7]. In ERP patients gradually increase their exposure to fear provoking situations, whilst resisting the urge to respond with a compulsion [8]. Despite treatment advances, individual differences in treatment acceptability and outcome persist. With respect to ERP, over a quarter of those commencing treatment disengage prematurely and of the remainder, 20% fail to benefit [8]. One theoretical framework, which may explain the variation in levels of treatment engagement and outcome in OCD, is the Common-Sense Model of Self-Regulation (CSM; [9]). The CSM posits that an individual’s behavioural responses and attempts to regulate a given health threat depend on their cognitive ‘representation’ of that threat (i.e. their ‘illness perceptions’; IP)[9, 10]. This representation comprises perceptions about the identity, consequences, causes, timeline and controllability of the illness [10].

The five types of IP identified within the CSM are often quantitatively assessed using the illness perceptions questionnaire (IPQ [11]), or its revised form, the IPQ-R (IPQ-R; [12]. The IPQ-R also assesses ‘illness coherence’, one’s overall understanding of the health threat and ‘emotional representation’, an individual’s affective response to illness such as anger and fear [12]. According to the model, the emotional representation drives attempts to regulate emotions. Studies using these measures have led to the establishment of a substantial evidence base, showing that IPs are associated with coping and patient outcomes across a range of health conditions, such as diabetes and post-heart attack [13].

Recently, attention has turned to whether the CSM is useful in understanding perceptions that underlie coping and outcomes in mental health conditions. A recent systematic review of studies in mental health found that the dimensions of IP identified within the CSM were endorsed by people with a range of mental health problems and that perceptions were associated with coping (e.g. support seeking) and outcomes [14].

However, the authors highlighted conceptual challenges when applying a model originally developed in the context of physical health conditions, to mental health populations [14]. Researchers have emphasised the importance of testing the validity of the CSM’s dimensions in mental health populations using open-ended questioning [15] or qualitative methodologies [14]. No study to our knowledge has used a qualitative methodology to explore the IPs of people with OCD. This study aims to use qualitative methods to identify and characterise the dimensions of IP in OCD. The findings will make it possible to evaluate the utility of the CSM in OCD and to ascertain whether any adaptation of the current model and its associated measures is needed. The identification and characterisation of IPs in OCD is an important first step in understanding whether unhelpful IPs lead to maladaptive coping and poorer outcome, providing insight into ways of enhancing or targeting management support for OCD.

## Materials and methods

The study received ethical approval from the NRES Committee North West—Lancaster (Ref: 13/NW/0506). All participants gave their informed written consent to take part in the study.

A semi-structured interview schedule (S1 File) was developed in collaboration with a person with OCD. The schedule consisted of seven open-ended questions (with follow up ‘probes’) exploring: OCD knowledge, symptom onset, current presentation, impact, coping methods and future expectations. The semi-structured format allowed the researcher the flexibility to explore new topics as they arose, eliciting any IPs not currently identified within the CSM or IPQ-R. Data collection took place in the UK over an 11-month period between 2013–2014.

Recruitment was conducted by two methods: a) open advertisement, including UK national OCD and anxiety charity websites and social media channels, b) invitation of participants in a multi-site OCD treatment trial (‘OCTET’) [16, 17]. Participants were eligible if they were age ≥16, spoke English, had met diagnostic criteria for OCD within the last 12 months (module G of the Mini-International Neuropsychiatric interview; MINI [18]). Individuals were excluded if they were currently experiencing psychosis or had organic brain disease. Though we did not automatically exclude those with a co-morbid condition, our protocol specified that individuals might be excluded if a co-morbid condition precluded their ability to discuss OCD objectively.

Participants expressing interest were given the option to take part face-to-face with a researcher (at their home or preferred location) or by phone. Interviews (including eligibility assessments) were undertaken by RP, a female researcher and PhD student with a background in psychology and with formal training and experience in qualitative research. RP received training in undertaking eligibility assessments using module G of the MINI as part of her role on the OCTET trial. RP had previously interviewed some participants recruited from the OCTET trial, but had no prior relationship to those recruited through advertisement. Consenting individuals meeting basic criteria for the study were administered module G of the MINI to verify OCD diagnosis. Participants recruited via the OCD trial did not undertake the MINI provided they had entered the trial within the past year, as this was undertaken as part of trial procedures. The maximum one-year period was chosen as the team agreed that participants’ experiences of OCD at a clinical level would be very recent and thus even if participants had started to recover, their perceptions would be relevant to understandings of current illness and consequently pertinent to the CSM.

All participants completed: a questionnaire collecting demographic and contextual information (e.g. duration of symptoms, other long-term conditions experienced), the symptom checklist from the Yale-Brown Obsessive Compulsive scale (Y-BOCS[19]) and the self-complete version of the Y-BOCS to measure OCD severity (total score range 0–40). Participants’ age, gender and symptom sub-types (as determined by the Y-BOCS checklist) were noted and monitored to check whether any change to the sample strategy was needed to ensure diversity in the sample. No change to the sampling strategy was needed as these characteristics showed natural variability. Interviews ranged from 27 minutes—2 hours 21 minutes and took place on a one-to-one basis, mostly within a single sitting, though two interviews were conducted over two sessions. Immediately following interview, notes were made on initial impressions and contextual points of potential relevance. Interviews were audio-recorded and transcribed verbatim. Transcripts were not shared with participants, but were verified for accuracy against recordings.

To preserve participant confidentiality, all participant data was labelled with a unique identifier and stored securely and separately to participant identifiable data (e.g. consent forms). Transcripts were anonymised immediately after transcription. Care was taken during the write up process to disguise any details that risked making participants identifiable.

### Analysis

Data were analysed using thematic analysis [20]. Transcripts were shared within the team (RP, KB, PB, AW) as recruitment progressed, facilitating group discussion around coding and emerging themes. When no new themes arose from the interviews, data collection ceased. After familiarisation with the data set, RP systematically coded each transcript using NVivo 10, taking into account the notes made after the interview. The notes helped to ensure the researcher could accurately recall the context and meaning of the interview. Data were coded both deductively (matching codes to the dimensions of the CSM) and inductively, to identify any new dimensions of illness perception that are not currently recognised within the CSM, or its associated measures, the IPQ-Q and IPQ-R. The codes were reviewed regularly and duplicates were merged on an on-going basis. Codes were reviewed and arranged into themes and sub-themes, with reference to whether concepts fell within existing dimensions identified by the CSM/IPQ-R, or were conceptually different and constituting entirely new themes. The integrity of this process was ensured by regular team discussion and agreement on themes and sub-themes. Feedback from participants on the study findings was not sought due to the geographical spread of participants.

## Results

**Fig 1:**
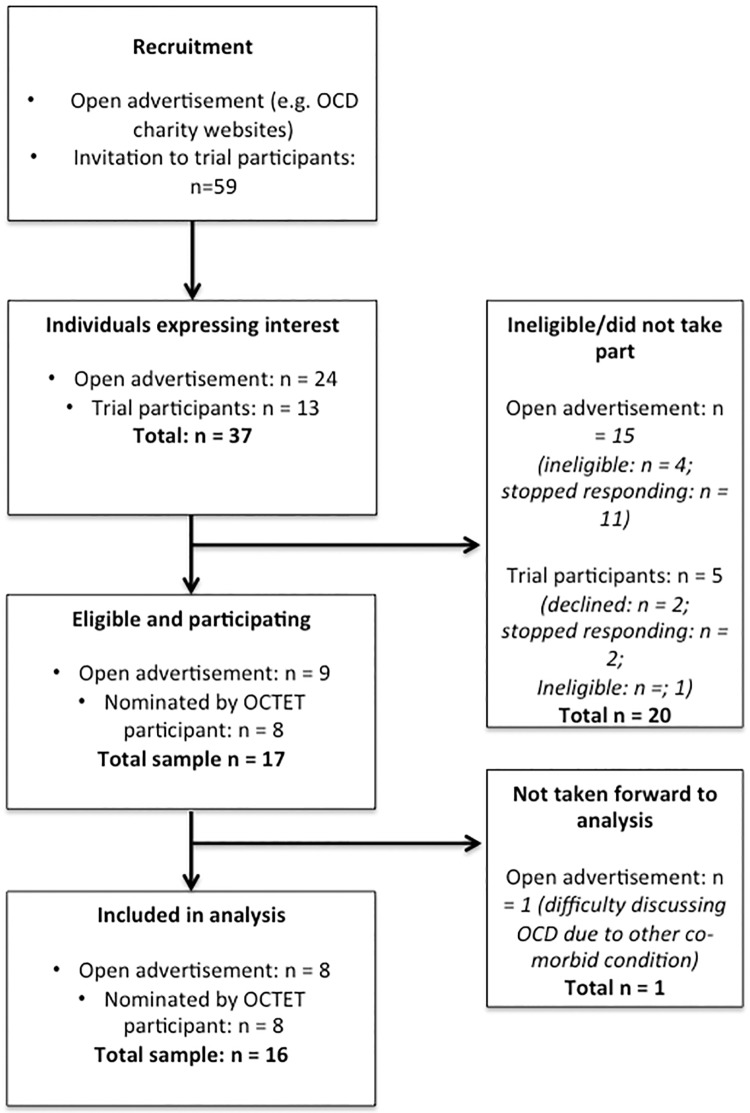
Flow of participant recruitment.

The flow of participants through the study is shown in in Fig 1. The characteristics of the sixteen interviewed participants are provided in Table 1. The mean Y-BOCS score of the included sample was 20.2 (7–29), indicating a ‘moderate’ level of OCD. Eleven participants reported an additional long-term physical or mental health condition, five of which suffered from more than one other condition. The most common of which was depression (n = 6), followed by anxiety (n = 3) and fibromyalgia (n = 2). All participants described their ethnicity as either White-British or White-Scottish.

**Table 1:** Characteristics of participants.

Participant ID	Interview setting	Gender M = male, F = female	Age at interview (years)	Time length experiencing OCD (years)	Received past psychological support for OCD CBT, counselling, other Y = yes, N = no
**1**	**Face to face**	**M**	**35–44**	**35**	**Y**
**2**	**Phone**	**F**	**55–64**	**30**	**Y**
**3**	**Face to face**	**F**	**45–54**	**26**	**Y**
**4**	**Face to face**	**F**	**25–34**	**5**	**Y**
**5**	**Face to face**	**F**	**35–44**	**15**	**Y**
**6**	**Face to face**	**F**	**16–24**	**14**	**Y**
**7**	**Face to face**	**F**	**25–34**	**15**	**N**
**8**	**Face to face**	**M**	**35–44**	**29**	**Y**
**9**	**Phone**	**F**	**45–54**	**30**	**N**
**10**	**Phone**	**F**	**45–54**	**12**	**Y**
**11**	**Face to face**	**M**	**35–44**	**31**	**N**
**12**	**Face to face**	**M**	**35–44**	**25**	**Y**
**13**	**Phone**	**F**	**25–34**	**18**	**Y**
**14**	**Face to face**	**M**	**16–24**	**6**	**N**
**15**	**Phone**	**M**	**35–44**	**20**	**Y**
**16**	**Face to face**	**F**	**45–54**	**10**	**Y**

Participants reported a wide range of obsessions and compulsions, with many individuals experiencing multiple symptom sub-types. Symptoms deemed by participants to be of greatest severity included obsessions about being responsible for harm to self or others (n = 4) contamination (n = 2), superstitious fears (n = 2) and compulsions around checking (n = 5), harm (n = 3), superstitious rituals (n = 2) and contamination related compulsions (n = 2).

The findings indicated support for all the dimensions of illness perception recognised by the CSM and its associated measures. Three additional themes that were deemed conceptually different to the dimensions of the CSM emerged from the inductive analysis: 1) Spectrum of OCD, 2) OCD is part of me and 3) OCD is reactive to internal and external influences.

### 1. Illness perception dimensions proposed by the CSM

Participants’ perceptions supported the following dimensions of the CSM and IPQ/IPQ-R, that is, perceptions about identity, cause, consequences, timeline, emotional representation, personal control/treatment control and coherence.

#### 1.1 Identity

**1.1.1 Applying the OCD label**. Though at the time of the interview, participants labelled their health problem as ‘OCD’, many indicated that this had not been the case earlier in the course of their condition. Indeed, participants often reported that it had taken many years to recognise OCD. In a small number of cases, the delay was related to perceiving their behaviours as ‘normal’ or fitting with their personality (e.g. someone who likes things ‘neat’), or even, in the case of one participant whose OCD started in childhood, due to family members perceiving symptoms as a transient developmental ‘stage’. The latter case highlights that the individual’s perception of their experiences might heavily rely on family members’ interpretations and actions, where the sufferer is very young at the point of onset. One participant believed that her mother’s insistence on excessive and meticulous care of the home during her upbringing, had led her to believe that “all *that* was normal.” The participant recalled that her ‘symptoms’ only started to become evident when she became older and started to spend more time with a partner, whose behaviour contrasted significantly to her own:

“*It probably then started hitting me that ‘This isn’t right that I’m doing these type of things’ because other people seem to be able to leave, you know, just be able to live with that, deal with that, without doing X, Y and Z….”*

(P.9, f).

More commonly, participants recognised that they were experiencing difficulties yet struggled to understand what the problem might be. Failed attempts to seek help from health professionals or family, lack of awareness of OCD (particularly where symptoms started at a time when OCD awareness was lower than today), or a failure to identify their symptoms as characteristic of OCD due to awareness of only the ‘common’ sub-types (e.g. ‘washing’ or ‘checking’), prevented people from our sample from recognising OCD. Instead, participants attributed their experiences to other mental health problems (e.g. generalised anxiety, depression) or to something ‘abnormal’ or ‘weird’ about themselves (e.g.“my quirkiness”), or even to having lost their grip on reality entirely:

“…*I honestly thought I was going mad*. *That I was losing my mind*, *losing control of my mind*. *And that it was in control of me and I wasn’t going to be able to stop it doing the things it wanted to do*. *Which was frightening*, *very frightening*, *terrifying…”*

(P.12, m)

It was unclear whether participants who attributed their symptoms to a ‘quirk’ or to having become someone that the “media portrays as mad”, saw their experiences as ‘symptoms’ of a health condition at all. Instead, their experiences seemed to be regarded as a result of a flaw in the self which they feared could make them liable to commit terrible acts. These individuals often expressed relief when later recognising OCD (e.g. through diagnosis), due to the realisation that their experiences could be explained by a ‘condition’:

“…*it was a bit of a light bulb moment for me really because…I just thought I was going mad*, *I didn’t know there was actually…this disorder if you like… had a name for it so that…I did feel a little better*, *I suppose*, *knowing that I wasn’t going mad and actually I had*, *you know that it was a mental health problem…”*

(P.2, f)

**1.1.2 The perceived symptoms of OCD**. At the point of interview, participants had identified their experiences as part of OCD for some time. Participants identified the hallmark symptoms of OCD; obsessions and compulsions. They described obsessions as unwanted, distressing thoughts or images (though images were discussed less frequently) that were difficult to control, ‘irrational’ and recurring:

“*I think obsession is a word that describes how your thought processes work*, *that you get locked in*, *you get brain lock*. *You go in a cyclical motion of something that you cannot…get it out of your mind*…”

(P.8, m)

Compulsions were recognised as excessive behaviours which were often repetitive, and performed despite not being truly necessary. Though participants most commonly discussed physical compulsions, mental rituals were sometimes described too. The following participant explained that his compulsions served a purpose of reducing uncertainty and making life easier, though acknowledged that this relief was short lived:

“…*there was a temporary resolution to the fact that if I did something [compulsion] then that would be okay*. *So if I checked the door multiple times*, *then I wouldn’t be three quarters of the way into town and think*, *‘oh I’ve got to go back*, *or I’d stay out and then worry about it the whole time until I got back…”*

(P.15, m)

Participants recognised that there was huge diversity in the ‘type’ of symptoms that people with OCD suffer from:

“…*it’s a very individual thing and it’s around obsessions of some sort and what people do to mitigate those obsessions, in again, just like a huge variety of different ways*…”

(P.15, m)

#### 1.2 Coherence

Through experience, personal research or therapy, participants developed a more coherent understanding of OCD. This included recognising their symptoms as a mental health problem but also wider aspects such as its treatment and possible causes:

“*Now I see it*, *I’ve read about it…I believe I can see it*, *exactly how it comes about*. *I can see it in other people*, *but it’s definitely a mental illness to me now*. *And I know I’m sure there’s very logical ways of treating it*. *And it’s definitely something*, *it’s totally understandable why people can get it*…”

(P.14, m)

Though individuals’ comprehension of their condition strengthened, some nevertheless struggled to make sense of certain aspects of OCD. Some spoke of their frustration as to why they continued to act on compulsions despite believing these behaviours to be excessive and irrational:

“*I know if I don’t check the door again*, *nothing is going to happen*. *I know at half 3 that I checked it at half 2 and it’s locked and it’s still going to be locked because there’s nobody else in the house*. *I’m not daft*, *I know that*, *but for some reason I have to go and do it again*”

(P.9, f)

Another participant reported that she was better able to manage obsessions for which she could deduce a logical explanation (worry about burning the house due to fear of hurting others), but felt puzzled as to how some obsessions had no logical origin, appearing to “come out of nowhere”:

“*I know that if I walk into* [place participant regards as contaminated] *it’s not really dirty*, *but I still feel like I'm being contaminated by something*. *So yeah*, *intellectually it makes…I can’t rationalise it but that emotional punch is still there*…”

(P.13, f).

#### 1.3 Cause

Participants rarely presented a fixed idea of what caused their OCD; instead, most offered a variety of possible causes, some of which they seemed to believe in more strongly than others. Participants often cited a combination of both ‘*internal*’ factors (e.g. the “connection in the back of your head”, “serotonin levels”, or genetics), and *external* factors, such as their upbringing (e.g. learnt behaviour), significant events or traumas. Internal causes could be biological or even the participant’s own personal characteristics, such as their way of thinking (e.g. someone who “thinks deeply”), their ‘character’ or ‘personality’:

“…*there is a particular group of people that are more susceptible to OCD because of their emotional make-up*, *their nature*, *the way they process thoughts*. *Erm… their moral sense of responsibility*…”

(P.8, m)

External or environmental causes of OCD also featured prominently within accounts. OCD was often linked to personal experiences, such as having ‘learnt’ or been instilled with behaviours regarded as OCD-like, during childhood:

“…both my parents have little quirks and I’ve picked them up and taken them to the next level, like they’re both very organised and rigid in how they like things…”

(P.4, f)

Other life experiences that contributed to the development of OCD included significant events that increased responsibility (e.g. birth of a child), experiencing or even hearing about a “scare story” (e.g. a gas explosion), or through trauma. One participant, who identified multiple potential causes of OCD, felt that a traumatic event had triggered her OCD:

“…*I didn’t know I was traumatised at the time*. *I didn’t understand that*, *but I think something went wrong then*. *I think something went*, *and put me into a state of alert*. *And I think since then*, *this alertness*, *this has caused me to try and be in control of everything*, *of every danger*…”

(P.3, f)

Sometimes, participants formulated personalised complex psychological explanations for their OCD’s development, which explained why circumstances or events may have led to them developing symptoms, for example, using OCD to provide a sense of control at a point where they had little control.

Running through many of these varied causes of OCD, was a common thread that OCD had originated from highly personal factors relating to the individual, such as their personality or an experience. This ‘individualised’ view of OCD was also evident in the way that participants acknowledged that causal factors might differ from person to person, depending on their particular set of circumstances:

“…some people develop it with a trigger. Like I can identify a trigger. Other people can’t, other people sort, it’s sort of been traits of it in their childhood. And it’s sort of escalated, things like that. Erm, or, I do think that you can be predisposed to it by the kind of temperament you’re born with…”

(P.3, f)

#### 1.4 Control

**1.4.1 Personal control**. Participants explained that early in the course of their OCD, they had struggled to understand how their symptoms could be controlled. Failure to recognise OCD led to an ever-increasing level of symptom severity:

“…*oh*, *I don’t like that cupboard*, *I’m just not going to use that cupboard*, *and then; oh*, *I don’t like that cupboard I won’t use that either*, *and it spread*. *Whereas knowing that now*, *every time I get it I can contain it more when it does happen now*, *so I suppose it’s because I didn’t resist at all*, *it just grew*.*”*

(P.4, f)

Participants such as this, who later developed an understanding of the need to ‘resist’ OCD, often seemed to have gained a sense of personal control through undertaking psychological therapy:

“…I suppose the exposure response prevention thing is the main one [treatment] and I don’t think anybody would do that off their own back because it’s sort of⋯Some of the things you’re supposed to do are incredibly scary and you wouldn’t think of doing that…”

(P.4, f)

In addition to resisting compulsions, some participants spoke of other methods of control, such as tolerating thoughts as opposed to ‘fighting’ them, or by trying to rationalise the thought. The following participant felt able to control her OCD to a degree, but explained that this took considerable personal effort:

“…I could just so easy get so bad again, if I didn’t mo-… if I didn’t keep my lid on it, well not keep a lid on it…but …if I didn’t try and stay in control of it if you like…so I have to…erm…y’know I have to constantly tell myself ‘No! You don’t need to wash your hands. No! You don’t need to wash your face’…”

P.2, f)

Other participants were aware that resisting their compulsions might be helpful, but felt unable to put this into practice, for example, due to not ‘accepting’ that they were becoming unwell, fear of increasing their anxiety or lack of confidence:

“I could probably do it (not check) if I knew that I was in therapy. I’m not confident, people tell me, my parents are saying “Can you try and do it yourself”, “no I can’t”, I’d have to have some sort of guidance. I’m not confident in doing it myself at all…”

(P.14, m).

This participant, who had not yet received psychological therapy for OCD, admitted that as a result, he simply tended to “get on” with his compulsions.

Occasionally, participants’ perception of personal control seemed to be increased by strategies that would not be recommended as part of modern exposure and response prevention based approaches, such as avoidance or by asking others for reassurance:

“*I rang my mum up the day before and went*, *‘do you think the world’s going to end tomorrow*? *She went*, *‘no’*. *I was like*, *‘thanks mum’*. *That’s all I needed*. *She’s very well trained…”*

(P.13, f).

Other techniques to ‘control’ OCD were based on individuals’ personal understanding of what made OCD better or worse, including both ‘reactive’ strategies aimed to manage the symptoms when they worsened (e.g. walking the dog to ‘calm down’), as well as preventative measures, aimed at managing symptoms. Some spoke of the importance of keeping ‘occupied’ or busy, for example through undertaking an enjoyable activity, reading a book or watching the TV. Two participants in particular seemed to be particularly proactive in keeping their OCD under control, taking steps to maximise their wellbeing through good self-care (e.g. eating and sleeping well) and strategies to promptly access treatment and manage pressure when symptoms started to worsen:

“…*I let the doctor know very quickly that this [increase in symptoms following difficult life event] is what was going on*. *I booked in myself into counselling very quickly that I accessed*. *I booked time off work to have a few days away*. *And because of all those steps that I took*, *I felt safe*…”

(P.8, m)

**1.4.2 Treatment control**. The majority of participants expressed a view that treatments such as CBT and pharmacological treatments had already or *could in the future*, improve their symptoms and that without treatment, OCD was unlikely to improve. Many perceived medications (e.g. antidepressants) as a useful treatment, which had helped to reduce their symptoms:

“..when I first started taking the tablets, I definitely felt more relaxed. Whether it’s just to get rid of that anxious feeling, because obviously when you’re not anxious, I believe I don’t check as much…”

(P.11, m)

There were often reservations however, about the sufficiency of medication as a standalone treatment for OCD. Some viewed medication as inferior to CBT, in that it was only ‘part’ of the solution, which failed to deal with the underlying problem:

*“…it [medication] helps with the symptoms and it does help*. *But it doesn’t really deal with the problem*, *so if you come off them all of a sudden it’s bad*…”

(P.14, m)

Psychological therapy often seemed to be favoured as a more acceptable and effective form of treatment:

“….you definitely need the therapy, I really don’t believe for one minute you would ever recover from OCD with just tablets…”

(P.5, f)

CBT was not the only therapy highlighted; some acknowledged that other therapies, such as mindfulness and transactional analysis had proven beneficial. Despite many perceiving treatments to be useful, a number of participants identified personal barriers that could hinder engagement with treatment or prevent therapy from being effective. Variation in the quality of therapist, or difficulty with continuity of treatment due to staff ‘turnover’, were seen as reducing the potential benefits of treatment. Personal beliefs also acted as a barrier to treatment, such as believing it not to be the right ‘time’ to receive therapy (e.g. being too stressed). One participant in our sample had never attempted to seek treatment for OCD. Although this individual spoke at length about OCD interfering with relationships, she concluded that her OCD was too ‘mild’ to warrant external support:

“…a lot of people it’s about anxiety and like err, worrying about contamination and illness and over-worrying about finding symptoms and things like that. I don’t, thankfully, I don’t have any of that. Which has always made me think that mine is pretty mild. Because it doesn’t affect me in that way, it’s just something that I really should deal with myself.

(P.9, f)

Whilst most acknowledged that treatments could be helpful, there was widespread doubt that their OCD could be ‘cured’. For participants who had suffered symptoms for extended lengths of time, the likelihood of being free of the condition felt remote:

“I’d like to think ultimately I won’t experience any of these problems, but I know that’s not going to happen…”

(P.15, m)

#### 1.5 Timeline

**1.5.1 Duration**. Participants held beliefs about the length of time for which they expected to experience OCD. Although some acknowledged that the duration might vary between individuals, the predominant view was that although their OCD could be improved, their vulnerability to OCD was permanent:

“*I think it’s always there*, *but you just have to deal with it*, *and the more you get control of it*, *it won’t interfere as much*…”

(P.6, f)

Sometimes a view of OCD as permanent seemed to derive from the person having already endured the condition for a long time period. Pessimism also seemed to relate to an inability to imagine their life without the condition:

“…I just find it really hard to think of me, ever being over it completely”

(P.2, f)

This participant felt that her condition had endured so long that it had become ‘ingrained’ and as such, permanent:

“…*after 30 years it’s become like a habit now and it’s very difficult to…it’s like breaking a habit I suppose*, *but it’s not a habit*, *I know that…it’s just ingrained in my psyche…”*

(P.2, f)

**1.5.2 Changing symptom severity and content**. Participants held views about the changing severity of their OCD. Many found that their symptoms waxed and waned over time. The following participant explained how his symptoms were increasing:

“…*I’m using about an hour to check in there [referring to a room in house]*. *I usually only spent about half an hour in there*, *everything else*, *there are other symptoms appearing*. *Erm*, *they’re just started to worsen gradually*…”

(P.14, m)

Some also discussed fluctuations on a more short-term day-to-day, or even hour-to-hour level. In addition to changing in severity, participants often explained that the content of their obsessions and compulsions shifted over time:

“…*through my life*, *it’s metamorphosised*, *it’s changed*, *it’s gone into different things*. *The only thing I’ve never had a problem with is symmetry*. *I’ve never had a problem hoarding*. *I’ve never had a problem going in and out of doors and things like that*. *But the checking*, *the contamination*, *the intrusive thoughts*, *all them things I have had*.*”*

(P.3, f)

#### 1.6 Consequences

**1.6.1 Experienced consequences**. Participants spoke about the level of impact that OCD had on their lives and in particular its interference with their ability to be productive and engage in enjoyable activities. Some felt that OCD had got in the way of their hopes, dreams and ambitions; for example, one participant had abandoned a career working with children due to a fear of committing abuse (despite seeing abuse as abhorrent). This fear had been compounded when her primary care physician (at that time) had misunderstood her difficulties and advised her to reconsider her career. One participant explained the broad impact of OCD on her life:

“…*it hasn’t ruined my life*, *but it’s made it extremely difficult and in areas*, *it’s in every aspect really*. *But it’s like employment*, *I’d love to be working*, *do a part-time job*, *but it ended up*, *I couldn’t go back*…

(P.3, f)

OCD also affected the lives of participants’ loved ones, creating strain on their relationships:

“…*He [partner] get’s very uncomfortable and he doesn’t like it*. *Erm*, *because he likes to do things his own way*. *He has to keep in mind*, *the things that he’s doing rather than*, *the things I want him to do*. *I guess*, *I nag*, *and I know I nag*, *which I don’t like doing…and he doesn’t like me doing*, *and I feel really bad*”

(P.7, f)

**1.6.2 Preventing consequences**. Although discussions tended to focus around the actual consequences of OCD, a sub-theme emerged around the feared consequences of OCD, which could occur if measures were not put in place to prevent them. Some were worried that through witnessing their anxiety and rituals, their children might develop a negative worldview, or might even develop OCD themselves. These individuals tried to prevent the feared impact by concealing their symptoms:

“*I didn’t want my children to be like me*. *I didn’t want them to pick things up like that*. *I didn’t want them to*, *although I couldn’t do it [conceal OCD] all the time*, *it’d be ridiculous to expect that I could*. *But of course*, *when they’re young*, *they don’t notice the same either*, *and you can get away with things*⋯

*(*P.3, f)

Rather than trying to hide their symptoms, one mother took a different approach to prevent her son from developing OCD, by trying to ensure that her son understood her behaviour to be excessive:

“..*I used to sometimes sit down with him and say ‘look*, *the reason why I do this is because*, *I want you to understand that it’s purely me*, *it’s not that everyone should behave like this’* ⋯”

(P.5, f)

One mother went further, explaining that her fear of others misunderstanding her OCD symptoms might lead to her losing custody of her children. To prevent this consequence, she was careful not to reveal too much about her condition to others.

Some participants were careful to ensure that the negative impact of their OCD was minimised as far as possible, particularly around friends and more distant family members, who might be offended by the requirements of the individual’s obsessions and compulsions:

“I’m very aware that if they knew how I was feeling it would really hurt them, so I’ve got to try and hide that from them. So, because the last thing they would want is to know that they were upsetting me, but I wouldn’t want them to know I was upset over something so trivial, as putting a book down on my table ⋯“

(P.9, f)

#### 1.7 Emotional representation

There was a sense of guilt amongst some individuals about the impact of OCD on other people. This perception led individuals to cast negative opinions on themselves. One participant explained that his fears about harm coming to his family had ironically led to him focusing more on himself:

“…its [OCD] made me into, quite a, not self-centred person, but I spend a lot of time on me. That’s not my natural disposition. Maybe it would have helped if…my self-esteem and confidence would have been a bit better. But it feels like I’m very selfish…”

(P.12, m)

Other participants feared shame, stigma, or embarrassment about their OCD. The following participant acknowledged that although his family members had never acted negatively towards him as a result of his OCD, he nevertheless struggled with these feelings:

*“…I felt embarrassed at times*, *I felt weak at times*. *I once spoke to my wider family… it’s not their fault*, *and it’s not anything they did*, *and it’s not that they were ever unsupportive*, *but it’s how I felt*, *and that needs to be understood*, *that it was my feeling not their*, *not what they said*, *but I sometimes felt like the black sheep of the family because I was the one with the mental illness*…”

(P.8, m)

Consequently, some kept knowledge of their OCD confined to a trusted group of close family and friends:

“…*there’s still a big stigma I suppose about it*, *about anything mental health*, *and I know it’s a lot better than it’s been before*, *but it’s still there…I think most people are more understanding now*. *But I don’t want people to know*…”

(P.10, f)

Indeed a small number had taken a significant amount of time to disclose their symptoms to health professionals or even to close family. For one participant, the degree of shame experienced was such that he concealed his OCD from his partner and health professionals for many years, leading to delays in help-seeking. In contrast to participants who spoke of relief when receiving diagnosis, the sense of shame continued after confiding in a health professional:

“*I was completely ashamed if I’m honest*. *I didn’t get a magical sense of relief [disclosing OCD]*, *which I was possibly hoping I would*. *After you think for so long that you can’t…’no I’m not going to tell anyone*, *not going to tell anyone’*, *and then it was only because of the state I was in that I actually said anything*…”

(P.15, m)

### 2. Emergent themes from inductive analysis

#### 2.1 Spectrum of OCD

A common perception amongst participants was that obsessions and compulsions were to some extent, ordinary phenomena experienced within the general population, e.g. “everyone has OCD really” (P.14). As such, there seemed to be a perception of OCD as presenting on a spectrum. As participants elaborated further however, most qualified that whilst such phenomena were common, the presentation in people reaching diagnostic criteria for OCD was more extreme, problematic and debilitating compared to those without a diagnosis:

“…I do believe that everyone has it [OCD], but whether or not you’d label that OCD, I don’t think so, I think that’s just normal life…there’s a point on that spectrum where it becomes abnormal, it becomes disordered, it becomes chaotic, erm, and it becomes life intrusive…”

(P.8, m)

Whilst the following participant shared a spectrum type view of OCD, he nevertheless believed that applying the label to behaviours in people without diagnosed OCD could cause offence:

“…I think everybody has a certain level of it [OCD] yeah. But I don’t think, I think that also demeans, like everyone is like ‘urgh, everyone has it a bit’, yes but it doesn’t impact their quality of life …”

(P.14, m)

#### 2.2 OCD is part of me

As discussed as part of the identity theme, some individuals did not initially label their experiences as OCD, sometimes confusing symptom with personality traits. At the point of interview, individuals were able to identify symptoms and applied the label of OCD. Despite this, there remained a prominent perception of OCD as entangled with individual’s perception of the ‘self’. In some cases, this perception seemed to have derived from experiencing the condition for long periods of time. For one participant, a perception of OCD as “ingrained” and unlikely to be fully treatable, led to an acceptance of OCD as a permanent part of his identity:

“*I’m just used to it*, *it’s part of me now*. *I’ve accepted it as part of me*, *which is probably a bad thing to do*, *but because I’ve had it so long*, *and I didn’t think there was any cure for it*…”

(P.11, m)

Sometimes, acceptance was so marked, that the individual was almost defiant in the need for others to similarly ‘accept’ their condition:

“…if I can deal with it and I have it all the time, then other people around me should be able to, and if they can’t, then I don’t think I really want them around me…”

(P.13, f)

In another case, the absorption of OCD into a person’s identity seemed to bring into question whether OCD was really an ‘illness’ at all. OCD was regarded as becoming part of the person’s personality and as such, the prospect of a complete ‘cure’ was almost out of the question:

*“In my mind, maybe there’ll always be the urge to; but yeah I could reduce my symptoms a lot; but not completely no….And that’s not a negative thing, it’s just the way people are. It’s just part of the way, it’s not a disease but it does become part of your personality. It becomes part of the way you think*.

(P.14, m)

It is worth noting that contrary to this, at other points in the interview, the same participant described OCD as a “mental illness”. This suggests that after diagnosis, some individuals may only partially conceptualise OCD as a health problem. The following participant described this as a source of cognitive dissonance:

“…*it [OCD] was something that lived in me but was separate from me*. *So again*, *it’s very paradoxical this sort of symbiotic relationship where*, *you know*, *it’s part of my identity so I need it and it needs me*, *otherwise it wouldn’t survive*, *but we are two separate things*.”

(P.13, f)

There was also uncertainty in determining which elements of behaviour were a result of OCD, and which were sub-threshold behaviours. One participant spoke explicitly about this difficulty:

“…it’s hard to think what is a symptom what is just me being a bit weird”

(P.4, f)

Another participant presented the opposite opinion, explaining that “I don’t think you lose all sense of what’s real.” This participant nevertheless seemed to express some uncertainty over whether she had recovered from OCD. Again, living with OCD for extended periods of time seemed to lead to a blurred boundary between having OCD and being without it:

“…I forget what it’s like not to have OCD, so I am trying to compare how I feel right now to how I felt when I didn’t have OCD and I’m not sure if I feel the same because it’s such a long time ago…so maybe, I am over it, maybe I’m not…”

(P.5, f)

Although participants believed OCD to be responsible for distressing and unwanted symptoms, some also saw it as able to manifest more positively in other aspects of their thinking and behaviour. One participant described how he “obsessed” over his hobbies and interests. Another explained how OCD helped her in her studies:

“…*part of it*, *I do like*, *like I said with [participant’s studies]*, *because it helps me see different points of view*, *and look into things*, *so I like that side of it*, *I just don’t like the horrible side* ⋯”

(P.6, f)

Indeed, this participant saw OCD as so integral to her personality that she expressed some reluctance in letting it go:

“…*even though I don’t like it*, *it does make me* ***me***, *so I would miss it a little bit*…”

(P.6, f)

#### 2.3 OCD is reactive to internal and external influences

As discussed in the ‘timeline’ section, participants saw their condition as changing in severity and content. In addition, participants held perceptions about the reactivity of OCD and the range of internal and external influences that shaped the condition’s presentation. Participants recognised that the content of their OCD (e.g. contamination) differed between individuals and often perceived the contents of their symptoms to reflect their own past experiences, their hopes, fears and current circumstances in life. One participant reflected on how his OCD had evolved as he had moved from childhood to adulthood and had begun to “*understand things more*”:

“…*I didn’t know what I was trying to prevent [checking the door handle as a child] other than the fact there was going to be a fire or something like that*, *whereas now it’s more about trying to prevent different bad things happening as like an adult…*”

(P.15, m)

Some participants identified a particular stimulus, such as reading about the need to keep their baby’s environment sterile, which had caused a new obsession or compulsion to develop. One participant explained that new obsessions and compulsions could arise from a single incident that had caused anxiety:

“I didn’t follow it up and check…by the time I got home I were like a dithering wreck. So I was in a negative mood all night, upset…I didn’t show it, but it was playing over and over in my mind. So then, after that once, I’ve had that negative feeling once and I won’t let it happen again, so that [checking] will be part of my routine then, in future…”

(P.11, m)

There was evidence that the sensitive nature of OCD led some people to question whether they were liable to pick up new symptoms from other sources. In one case, this prevented a participant from engaging in group-treatment, due to fear of this introducing her to other types of OCD:

*“…they have a lot of depression support groups and things*, *it’s knowing other people might help*. *I’d worry about picking up other people’s obsessions though*, *not contagious but*, *oh*, *I haven’t worried about that until now…*”

*(*P.4, f)

In addition to seeing the content as vulnerable to change, participants identified various influences that affected the severity of their OCD. Over the short-term, participants believed OCD could be triggered by particular contexts or situations, which exposed the individual to their feared situation. Triggers were diverse and related to the individual’s particular ‘type’ of OCD, for example, one participant reported that reading or hearing about dangers in the media triggered obsessions about harm. One participant who experienced a fear of making a mistake at work explained how her symptoms increased and decreased in line with her daily activities:

“…*I was on my way to work thinking ‘oh my god’–obviously not so much now*, *but that was probably the worst time of day*. *The best time is I’ve done*, *I’ve got everything which I consider important out of the way*, *done*, *normally by mid-afternoon…then I can start to relax a bit…and the symptoms used to come down*…”

(P.5, f)

Conversely, some found that there were situations where their symptoms improved; for example, when on holiday. The following participant explained that her symptoms decreased when visiting a family member’s house, as she felt less responsible for untidiness:

“…*I can shrug it off as ‘their mess’*. *I can rub it off as their issues and I’m fine with it…I don’t feel the need to go in and start sorting out all their stuff…*”

(P.9, f)

Aside from triggers that related *directly* to the content of the individual’s obsessions, participants perceived a wide range of life pressures as affecting the course of their OCD. Such pressures included life events such as relationship breakdowns and deaths, as well as on-going stressors like work pressures and family illness. One participant explained that the accumulation of stress over time led to his current OCD symptoms being particularly severe:

“…*it’s worse when I’m tired and stressed*, *particularly around stress*. *I’m not just talking about stress at work*. *If I’m stressed then in general*, *then the OCD behaviours and thoughts become worse and more frequent*⋯*”*

(P.15, m)

Conversely, this participant recalled that his symptoms had reduced when he had been studying at university:

“…*I think it was probably because I was relaxed and the pressures…I know university’s got its own pressures*, *but I was away from home for the first time and people weren’t expecting me to do some of the things around neatness*…”

(P.15, m)

Some participants explained that it was not just unpleasant life pressures that caused OCD to worsen, but positive ones too. Several participants, both male and female, talked about experiencing an increase in symptoms following the birth of a child, due to the increased sense of responsibility:

“*I’ve experienced OCD as a result of trauma*, *good and bad trauma*, *and that sounds a bit bizarre but having children*, *when I’ve had children*, *and suddenly I’m responsible for this young life*…”

(P.8, m)

As well as affecting OCD over the longer term, participants described short-term pressures, which worsened their symptoms. There was a perception that symptom levels increased and declined in line with levels of anxiety throughout the day. The following participant explained how day-to-day stressful events could lead her anxiety levels to increase and ultimately OCD to worsen:

“*I can be*, *just generally anxious about getting somewhere*, *or getting to an appointment*, *to doing something on time*. *That can make it worse*. *You know*, *it’ll take me longer to check*, *and I’ll have to go back more*, *you know it’ll take me longer to get out of the house*…”

(P.3, f)

Internal influences were also deemed to affect OCD severity. Some perceived that certain factors depleted their personal resources to fight OCD (low mood or tiredness), leading to an increase in symptoms. Other internal influences on severity included current physical illness and hormones, the latter of which was a perception specific to females.

## Discussion

This study aimed to identify and characterise the IPs of people with OCD. All dimensions of illness perception described in the CSM (and IPQ/IPQ-R) were evident within the data, suggesting that there are parallels in the way that people with physical illnesses and OCD perceive their conditions. As has been found for physical illnesses, participants held perceptions about the label and symptoms of their condition, their ability to control it, its causes and consequences, their emotional responses to the illness, the likely time course and the degree to which they had formed a coherent understanding of their condition. Despite all illness perception categories being evident in the data, there were however, differences in the way individuals perceived OCD compared to how individuals might perceive a physical illness. Inductive analysis revealed three novel aspects of illness perception which supplement the CSM dimensions, relating to how people see OCD as ‘part’ of them, the factors that influence the severity and shape the appearance of OCD (i.e. its sub-types), and a perception of the disorder presenting as a spectrum in the general population. The emergence of these three novel aspects suggests that in mental health conditions (specifically in OCD), there is an added layer of complexity in our understanding of ‘illness’. It is important to note however, that there is some conceptual overlap between the new themes presented and those within the CSM. For example, the proposed theme of the ‘reactivity’ of OCD could be deemed to add information to perceptions of the degree to which the illness severity fluctuates (timeline cyclical). What seems to be novel here is the importance that participants placed on the influences that were perceived to affect OCD severity *and* content. Perceptions of the *nature* of these influences appeared to be important, for instance, a belief about the possibility of ‘picking up’ additional OCD obsessions from others reducing an individual’s willingness to attend a support group. The ‘timeline’ sub-scales of widely used measures of illness perception, the IPQ-Q and IPQ-R, do not include any questions that capture perceptions of potential influences on the condition. Therefore, whilst there was indeed evidence that the existing dimensions of the CSM (and subsequently the IPQ-R) usefully capture sufferers’ perceptions of OCD, we would argue that the additional themes presented here contribute to a fuller picture of participants’ illness perceptions.

Although our sample were able to reflect on OCD as a condition, there was evidence that some did not hold distinct ‘illness perceptions’ early in the course of the disorder. Indeed, accounts suggested that some participants initially failed to see their experiences as ‘symptoms’ of a health problem at all. Similarly, in physical illness, it has been proposed that we use ‘heuristics’ to decide whether a symptom is indicative of a health condition or alternatively the result of an age-related change or a temporary reaction to a stressor, for example [21]. However, there were indications that this delay in illness recognition was in some cases prolonged, exacerbated by barriers in obtaining a diagnosis and seeking help. Barriers such as parents’ dismissal of OCD symptoms as a passing phase, or ‘normalisation’ of OCD-like behaviours as part of upbringing, led to OCD not being recognised for many years. It seems possible that views of OCD as on a ‘spectrum’ might be linked to such interpretations. Further obstruction to recognising OCD was created where individuals perceived a problem but attributed their experiences to a ‘fault’ of the self, rather than a condition; for example believing symptoms to be a ‘quirk’ of personality, or even that they had transformed into, as one participant put it, a ‘monster’. These findings, together with other reasons for delayed help-seeking identified in the literature (e.g. stigma[22]), offer insight into the reasons for the substantial delay in help-seeking observed in OCD [23]. The findings suggest that the conceptualisation of OCD as a health problem is a crucial step necessary in initiating appropriate health related behaviour, such as seeking treatment through the medical system including psychiatry or clinical psychology. Such delays may serve to worsen the severity of OCD. For instance, in the case of someone with fears of becoming a child abuser, a lack of alternative explanation for these thoughts (i.e. a mental health condition) would likely allow this highly distressing view of themselves to grow. Unsurprisingly, participants often expressed relief when they realised that a mental health condition could explain their symptoms. Our findings highlight the need for medical professionals (particularly those who act as first point of contact such as general practitioners) and the general public to be better educated about OCD and particularly, for awareness campaigns and professional education to extend knowledge beyond ‘washing’ and ‘checking’, to the less widely known sub-types of the disorder.

With respect to the coherence dimension of illness perception, although participants had often gained an understanding of the condition following diagnosis, and through treatment, they experienced a paradox in that they continued to perform compulsions despite believing these behaviours to be excessive or ‘irrational’.

This quandary might be particularly marked in OCD due to the fact that some symptoms of the condition are not as clearly experienced by the individual against their will (as in physical health conditions), but are in themselves, deliberate actions (i.e. compulsions) performed by the sufferer. Clearly, in a condition where some of the symptoms are acts that are determined by the sufferer, understanding of the illness takes on a different meaning than in physical conditions where symptoms are more clearly experienced as “happening to” rather than “done by” the sufferer.

Participants perceived varied and often serious consequences of their OCD. OCD interfered with people’s ability to function at work, their relationships, as well as their social and leisure activities. In addition to this, there was a distinct sub-theme around the feared impact of OCD; here there was a particular focus on the possible impact of OCD on those around them, such as a fear of their children ‘picking up’ OCD by witnessing their behaviour. This perception led to added pressure for individuals who went to significant lengths to *prevent* the potential impact of OCD on others, for example by concealing their OCD from their children over extended periods of time. Many appeared to have persisted with this concealment with little professional guidance as to the helpfulness of this strategy. This finding highlights the need for treatments and support in OCD to not only focus on the family working towards improving the individual’s symptoms, but to help the sufferer to manage their condition in a family context.

The finding that individuals saw OCD as ‘part’ of themselves emerged spontaneously from the data and had not been prompted by the interview schedule. Parallels can be drawn between the findings presented here and those of other qualitative studies exploring illness perceptions in mental health conditions. Kinderman et al [24] found that individuals with *current* psychosis struggled to discuss their psychosis as a separate entity, incorporating unusual beliefs (which might be considered ‘delusions’) within their descriptions of their condition. These findings led the authors to question the applicability of the CSM to psychosis, as the fusion between the condition and the self was such that the authors concluded that people with current psychosis may not hold ‘illness beliefs’. However in this case, some participants *explicitly* acknowledged their perception of OCD being ‘part’ of themselves. It could be argued that the extent to which illness is seen as a part of the self or separate from the self is an additional dimension of illness perception.

Many participants expected the timeline of their condition to be long (often permanent) and were pessimistic about the chance of their condition fully remitting, even with treatment. Findings from the physical health literature indicate that experience of a long duration of illness, which in our sample extended over the course of years or even decades, is associated with expectations of a long timeline [10]. As Leventhal points out, these beliefs are not likely to be based on a single attribute but on a combination; for instance, having received treatments which did not ‘cure’ the condition [10]. The findings presented here suggest that perceptions of the link between OCD and the self might constitute a further perception that contributes to the sense of illness permanency. These findings were echoed by a study of family members perceptions about OCD [25]. Given the known efficacy of treatments for OCD in the form of CBT with ERP as well as pharmacological treatments [5, 6], such pessimistic views may be unwarranted. Furthermore, in the context of a condition in which treatment depends heavily on personal efforts to challenge the condition (e.g. carrying out ERP), low expectations for recovery could lead to poor engagement or even abandonment of treatment. Since treatment engagement is a particular challenge in this population, this may be a salient finding [8].

Related to the perception of OCD as part of the self, some participants saw less problematic ‘symptoms’ of OCD as personality traits that existed alongside other clearly unwanted and distressing symptoms. In some cases, these less problematic ‘symptoms’ were even regarded as positive. In rare cases, individuals even described some reluctance to fully let go of their OCD. Though it should be acknowledged that these participants might have attempted to identify some ‘positive’ aspects of OCD in order to help them cope with the condition, it is also possible that this perception was partially fuelled by the view of OCD as presenting in a spectrum in the general population, whereby symptoms can be exhibited without disability or distress. It was notable that findings from a recent qualitative study investigating the acceptability of two CBT based interventions for OCD also identified that positive perceptions of OCD created unhelpful ambivalence about treatment, which could acted as a barrier to engagement [26]. Given that personal qualities and abilities would not be seen as part of OCD according to diagnostic criteria, it might be argued that the root of their apparent ambivalence might be due to a misunderstanding of what OCD is as a condition, that is, confusing the condition with *who* they are as a person. To add to this ambivalence, whereas symptoms of physical illness are clearly unwanted and do not bring benefit to the individual, people with OCD continue to perform compulsions due to the (albeit short-term) relief that they bring to them. As such, commitment to ‘change’ might be inhibited by a degree of internal conflict as a result of the ambiguous presentation of ‘symptoms’ in OCD as unwanted, yet intentionally performed. Whilst further longitudinal research is needed to test whether perceptions of OCD as part of the self lead to pessimism and reduced treatment engagement, it seems possible that this perception might need to be overcome at the early stages of treatment.

The findings presented here also suggest that in OCD, individuals see their condition as highly personal and individual to them; it emerges from vulnerabilities in their own character and from their personal life experiences. OCD presents in personalised forms, coloured in content (or symptom sub-type) by events in their life and their personal hopes and fears, and then reacts and evolves on a continuous basis, in response to psychosocial stressors and life events. It seems likely that seeing the condition as individualised would make the sufferer uniquely connected to their condition in a way that perhaps someone would not be in physical illness, thus contributing to the perception that it is ‘part’ of them.

The high degree of reactivity of OCD highlighted here suggests that people are acutely aware that their condition will respond to their actions and experiences in life. There was some evidence that this awareness grew with experience of OCD and helped people to develop ways of coping with the disorder, such as taking steps to manage pressures. This awareness did however on occasion seem to breed problems of its own, for example where individuals expressed concern about meeting other people with OCD due to a fear of their OCD taking on other people’s symptoms. This may in itself perpetuate symptoms as people avoid opportunities for support or even perhaps avoid talking about their OCD.

### Strengths and limitations of study

As our sample includes individuals who have actively volunteered to take part in a study about OCD, these individuals may not be representative of the wider population of people with OCD. Participants’ willingness to discuss their experiences with a researcher may indicate that they are more open or hold different beliefs about OCD than others who did not take part. That said, the discussion of feelings of shame, embarrassment and need for concealment within the interviews suggests that participants who were not usually open about their OCD did put themselves forward for interview. Our sample included participants who experienced an additional physical or mental health condition alongside OCD. Though it is possible that these additional problems affected participants’ perceptions of OCD, OCD is known to frequently co-occur with other conditions, particularly with depression [27]. This means that our sample may be more reflective of the wider OCD population.

Due to resource constraints, it was not possible for two researchers to code the entire dataset. Though this is a limitation, we increased rigour by sharing the transcripts within the team and holding discussions about emerging codes and themes as recruitment progressed. The wider team were also involved in finalising the final set of themes and sub-themes.

Participants sometimes discussed experiences that had taken place some time ago and were talking retrospectively about the way they had thought and felt. Participants’ experiences of coping with OCD since this time, particularly in cases where participants had received therapy, may have coloured these memories. Though we cannot be confident about the accuracy of these recollections, it would be difficult to prospectively identify individuals at early stages, especially as we know many delay diagnosis or treatment seeking for some time. It is also possible that insights gleaned from this study, particularly about early experiences of help-seeking (e.g. medical professionals failing to recognise OCD), might be less valid today, given that participants were often discussing a considerably earlier time. Failure of medical professionals in diagnosing OCD has however been recently reported elsewhere [28].

Recruitment of treatment seeking individuals (from the trial) may have resulted in our sample holding particularly favourable views about psychological therapies. Whilst we cannot discount this possibility, our inclusion of participants from other online sources might have helped to mitigate this bias. Indeed, our sample included one participant who had never sought treatment.

The involvement of a person with lived of experience of OCD is a strength of this study as it helped to ensure that the schedule was likely to elicit discussions that were important to people with OCD, as well as ensuring the questioning was acceptable. Unfortunately, we did not involve a person with lived experience of OCD in analysis; such involvement may have yielded additional insights about perceptions of OCD that were not recognised by the study team.

### Conclusion

Application of a theoretical model derived from the physical health literature to OCD has garnered important implications for the effective management and treatment of OCD. For successful treatment, people with OCD must navigate a system of healthcare that is built around physical health conditions. Within this model, initial attempts to seek treatment depend on a conceptualisation of experiences as ‘symptoms’ of a condition. The findings presented here suggest that help-seeking for OCD can be impeded by a perception of experiences, not as symptoms, but as manifestations of personality. There is an urgent need to educate the general public and professionals to gain an understanding of the wide and varying presentations of OCD. People with OCD may benefit from support which disentangles the condition from the ‘self’ and which provides optimism around treatment. In addition, support for people with OCD should address sufferers’ concerns about how the condition affects their family members and the best way to manage its impact on their loved ones. Future studies are now needed to test whether the perceptions of OCD identified here affect coping and outcome.

## Supporting information

S1 File Participants with OCD interview schedule. (DOCX)
