# Supplementary material for: Illness perceptions in people with obsessive-compulsive disorder; A qualitative study
Source: PLoS One. 2019 Mar 20;14(3):e0213495. doi: 10.1371/journal.pone.0213495 (PMC6426201; doi:10.1371/journal.pone.0213495)
Supplement: S1 File — (DOCX) [file pone.0213495.s001.docx]

Participants with OCD interview schedule

| **Questions** | Prompts |
| --- | --- |
| **1) What do you know about OCD?** | Terminology? Duration? Do you know how it should be treated? Who? i.e. are some people more vulnerable than others? Different types of OCD? Has your knowledge changed with time? How/why (e.g. onset of being unwell) |
| **2) Tell me about how your symptoms of OCD started?** | When did you recognise OCD? What led up to it? Cause? What did you do? How did you react? Looking back, were there signs before? |
| **3) What are your symptoms like at present?** | Do symptoms fluctuate? If so, what improves symptoms/worsens symptoms? E.g. treatment. Do relatives’ actions affect you? Any triggers? Contrast how this week is compared to previous weeks. |
| **4) Tell me about a typical day in your life.** | How does OCD affect your life? Practically (lifestyle change) & emotionally (how do they feel about it). Any positives? Relationships with others/family? What about other family members? Does this fluctuate? Is this important to you? |
| **5) How do you deal with the symptoms you’ve just described?** | Has this changed with time? Why/how? |
| **6) Tell me what the future looks like at the moment?** | Stay the same, improve/worsen? Will circumstances/impact on you differ in future? Do you think it can be ‘cured’? |
| **7) Do you think there is any form of support that does/would help you live with OCD?** |  |
